# Anlotinib in Chinese Patients With Recurrent Advanced Cervical Cancer: A Prospective Single-Arm, Open-Label Phase II Trial

**DOI:** 10.3389/fonc.2021.720343

**Published:** 2021-11-02

**Authors:** Jun Zhu, Chunyan Song, Zhong Zheng, Lingfang Xia, Yanqiong Chen, Guihao Ke, Xiaohua Wu

**Affiliations:** ^1^ Department of Oncology, Shanghai Medical College, Fudan University, Shanghai, China; ^2^ Department of Gynecologic Oncology, Fudan University Shanghai Cancer Center, Shanghai, China; ^3^ Department of Radiology, Fudan University Shanghai Cancer Center, Shanghai, China

**Keywords:** anlotinib, VEGF inhibitor, monotherapy therapy, recurrent advanced cervical cancer, cervical cancer

## Abstract

**Objective:**

This phase II, single-arm, prospective study aimed to evaluate the efficacy and safety of anlotinib in Chinese patients with recurrent or metastatic cervical cancer (CC).

**Methods:**

Patients with histologically proven recurrent or metastatic advanced CC were enrolled at Fudan University Shanghai Cancer Center. Patients received 12 mg of oral anlotinib daily before breakfast for 2 weeks of each 3-week (21 days) cycle separated by a 1-week interval. Anlotinib was administered orally until disease progression, patient withdrawal, intolerant toxicity, or death. The primary endpoint was the objective response rate (ORR) according to the Response Evaluation Criteria in Solid Tumors, and the secondary endpoints included the disease control rate (DCR), progression-free survival (PFS), overall survival (OS), and safety.

**Results:**

Between September 2018 and November 2019, 41 patients were recruited. The median age was 53 years old. The histological results revealed that 82.9% of the recruited patients had squamous cell carcinoma, 14.6% had adenocarcinoma, and 2.4% had other types. At the data cutoff date, six patients were still being treated, and 35 patients had discontinued treatment. Forty (40/41, 97.5%) patients were evaluated for treatment response. The median PFS and OS was 3.2 and 9.9 months, respectively, in patients who received anlotinib treatment. The ORR was 24.4%. In addition, 34.2% (14/41) of patients were confirmed to have stable disease, and 39.0% (16/41) of patients were confirmed to have progressive disease. The DCR was 58.5%. Ten patients (10/41) had a confirmed response during the follow-up period. Most adverse events (AEs) were grade 1 or 2. High-grade AEs (grade 3) included urinary leukocyte positivity (9.8%), hematuria (4.9%), and hypertension (2.4%).

**Conclusion:**

This is the first study to evaluate the efficacy and safety of anlotinib in Chinese patients with recurrent or metastatic CC. Anlotinib produced durable clinical responses with manageable safety in these patients.

## Introduction

Cervical cancer (CC) is one of the most common gynecologic cancers, ranking fourth in morbidity and mortality among women worldwide (1). It is reported that the incidence of CC ranked second only to breast cancer among Asian women (2, 3). The recent statistical data illustrated that globally, there were 528,000 new cases of CC in 2018 and 311,000 CC deaths (1). In China, new cases account for approximately 20% of CC cases, and the number of cases still increases annually. For patients who develop recurrent and advanced CC (stage IVB), it is difficult to obtain clinical benefits through radical excision and regional radiation once recurrence or metastasis occurs (4). The guidelines of the National Comprehensive Cancer Network (NCCN) and European Society of Gynecological Oncology (ESGO) recommend palliative chemotherapy as the standard treatment for patients with recurrent and advanced CC (5, 6). However, the reported response rate for second-line or later therapy is relatively low at 15%–30%, and the duration of response is short in recurrent or metastatic CC (7, 8). Therefore, novel targeted therapies with low toxicity are anticipated for recurrent or refractory CC.

In recent years, with the development of molecular targeted therapy, anti-angiogenic therapy has been used in the treatment of CC (9). Anti-angiogenic therapy not only enhances the synergistic effect of chemotherapeutic drugs and reduces the adverse effects of treatment, but it also has important significance for improving clinical efficacy and treatment compliance (10, 11). A large number of clinical studies have confirmed that high expression of vascular endothelial growth factor (VEGF) occurs during the development of a variety of malignant tumors, which may be related to biological behaviors such as invasion and metastasis, leading to poor prognoses (7, 12, 13). CC is closely linked with human papillomavirus (HPV) infection, and an *in vitro* study revealed that HPV participates in the regulation of VEGF–vascular endothelial growth factor receptor (VEGFR) pathways by upregulating the expression of VEGFR2, thereby causing tumor angiogenesis (14). Targeting VEGF can therefore be used as an important target for molecular targeted therapy to prevent tumor angiogenesis. Bevacizumab, a VEGF inhibitor, was approved by the US Food and Drug Administration in 2014 to be used in combination with chemotherapy for patients with relapsed or advanced CC (15). Clinical studies of bevacizumab have revealed that anti-vascular therapy is useful for patients with recurrent or metastatic advanced CC (16). However, the high cost and severe side effects of bevacizumab limit its wide use in developing or undeveloped areas.

Anlotinib is a new type of oral multi-target tyrosine kinase inhibitor (TKI). It can effectively inhibit VEGFR, platelet-derived growth factor receptor (PDGFR), and fibroblast growth factor receptor (FGFR), c-Kit, and other kinases that inhibit tumor angiogenesis and tumor growth (17). A phase III trial of advanced non-small cell lung cancer confirmed the effectiveness and safety of anlotinib (18). Additionally, phase II clinical trials of anlotinib treatment for medullary thyroid carcinoma, soft tissue sarcoma, and renal cell carcinoma have been completed (19–21), and more phase III trials are scheduled to be completed in the near future. However, anlotinib has not been evaluated in patients with recurrent or metastatic advanced CC. Therefore, we conducted this phase II study to evaluate the efficacy and safety of anlotinib in patients with recurrent or refractory CC.

## Materials and Methods

### Study Design and Patients

This was a prospective, single-arm, single-center, exploratory phase II clinical study aimed at evaluating the effectiveness and safety of anlotinib hydrochloride in the treatment of recurrent advanced CC. The study protocol and all amendments were approved by the institutional ethics committees of Fudan University Shanghai Cancer Center (ChiCTR1800020116). All patients provided informed consent. The research process strictly followed the guidelines of the China Food and Drug Administration Clinical Drug Clinical Trial Quality Management Practices and the Declaration of Helsinki.

The criteria for inclusion were as follows: age ≥ 18 years old; histologically confirmed metastatic, recurrent, or persistent CC; an Eastern Cooperative Oncology Group performance status (ECOG PS) (22) of 0–2; treatment with at least one line of platinum-based chemotherapy; no prior treatment with VEGF inhibitor; documented radiological progression during the last treatment administered before enrollment; there were no previous heart, liver, kidney, brain, or hematopoietic system diseases; an expected survival time of ≥3 months; and measurable disease according to the Response Evaluation Criteria in Solid Tumors (RECIST) version 1.1 (23).

The criteria for exclusion were as follows: other malignant tumors present; pregnancy or breastfeeding in women; any factors that affect oral medication use (such as an inability to swallow, chronic diarrhea, or intestinal obstruction); any bleeding event with a severity grade according to the National Cancer Institute Common Terminology Criteria for Adverse Events (NCI-CTCAE) (24) of 4 or higher in the 4 weeks before screening; uncontrolled high blood pressure; history of unstable angina or abnormal blood clotting function; arterial/venous thrombosis within 1 year before screening; and a history of immunodeficiency.

### Procedures and Assessment

Patients were enrolled between September 2018 and November 2019 at Fudan University Shanghai Cancer Center. Anlotinib was administered at a daily dose of 12 mg orally on days 1–14 of each 21-day cycle. Adverse events (AEs) and laboratory abnormalities were classified and graded according to NCI-CTCAE version 4.0. The dose of anlotinib could be reduced to 10 or 8 mg/day in patients with grade 3 or 4 treatment-related toxicities. All cases were followed up to disease progression or death.

Responses were assessed by investigators according to RECIST version 1.1 using computed tomography (CT) or magnetic resonance imaging (MRI) at baseline and every two cycles thereafter (6 weeks). The primary endpoint was the objective response rate (ORR), which was defined as the proportion of patients with a complete response (CR) or partial response (PR) according to RECIST 1.1. The secondary endpoints included progression-free survival (PFS), overall survival (OS), disease control rate (DCR), and safety. The PFS and OS were defined as the interval time from the completion of screening with eligibility confirmed to the progression of disease or death, respectively. The DCR included CR, PR, and stable disease (SD).

### Statistical Analysis

A Simon’s optimal two-stage design with a one-sided α of 0.05 and power of 0.90 was performed. The sample size was calculated using the null hypothesis that the ORR was 5% or lower (H0 = 5%) and the alternative hypothesis that the ORR with anlotinib would be 20% or higher (H1 = 20%). After testing the drug in 21 patients in the first stage, the trial would be terminated if one or fewer responded. If the trial went on to the second stage, a total of 41 patients would be studied. Overall, if the total number responding was less than or equal to four, the study would be terminated.

The 95% confidence interval (CI) was calculated using Clopper–Pearson method. The Kaplan–Meier method was used to estimate PFS and OS with the associated 95% CI. *Post-hoc* analyses were conducted to explore prognostic factors for the treatment with anlotinib in the recurrence or metastasis of cervical cancer, and to assess the hazard ratio (HR) and the 95% confidence interval (CI). All statistical analyses were performed using IBM SPSS 22.0 software.

## Results

### Patients

**Figure 1:**
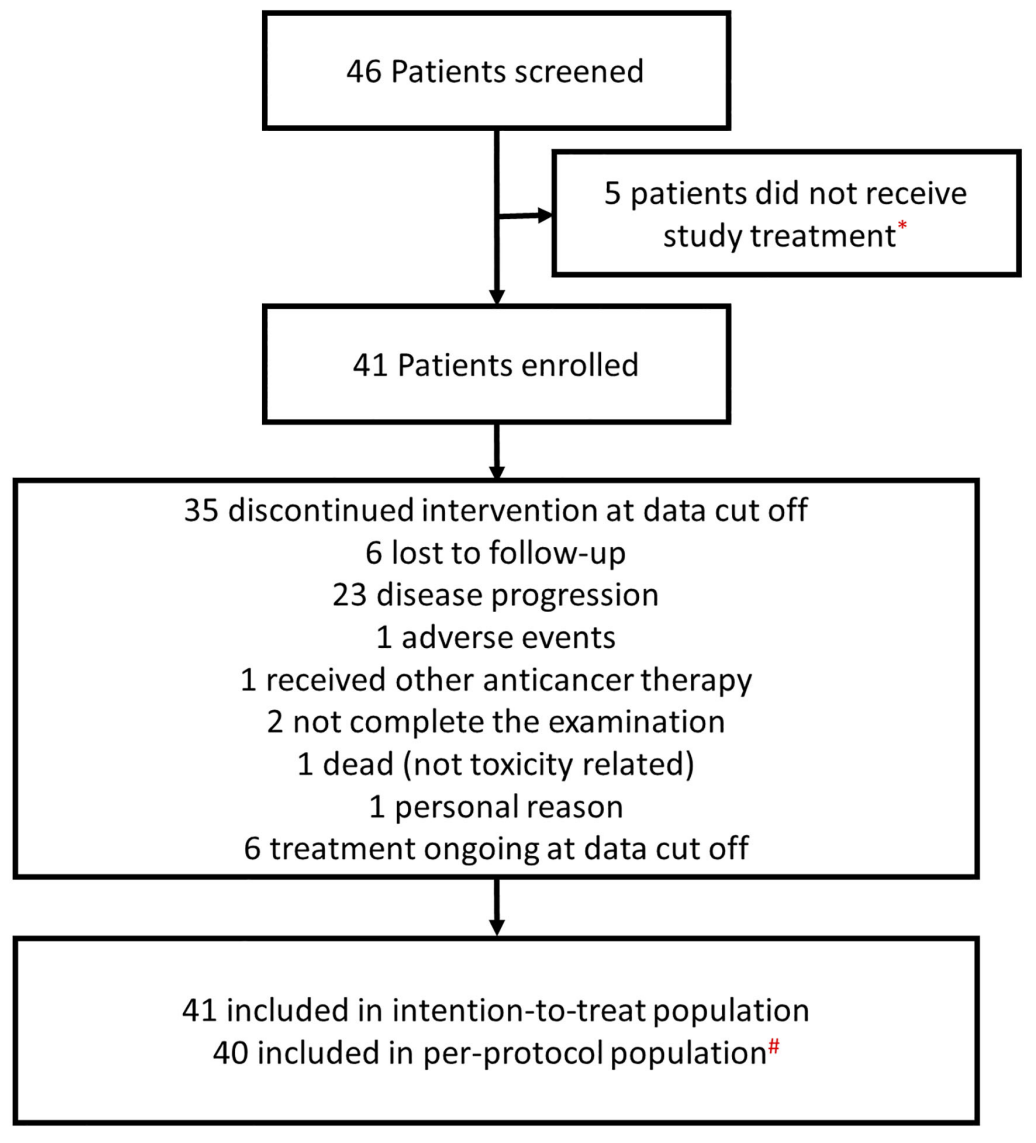
A flow diagram of patients enrolled in the study. *Five patients did not initiate the study because the eligibility criteria were not met. ^#^One patient was excluded from the per protocol population sinceno efficacy assessment was performed yet.

From September 2018 to November 2019, a total of 46 patients were screened, of which 41 patients with recurrent advanced CC were enrolled into this study. A flow diagram for patients consented for the study is shown in **Figure 1**. Five patients did not initiate the study because the eligibility criteria were not met. One patient was excluded from the per protocol population since no efficacy assessment was performed yet. The baseline characteristics for patients are summarized in **Table 1**. The median age was 53 years old (range: 42 to 64 years). The histological results showed that 82.9% (34/41) of recruited patients had squamous cell carcinoma, 14.6% (6/41) had adenocarcinoma, and 2.4% (1/41) had other types. Forty patients (97.6%; 40/41) had a ECOG PS score of 0, and only one patient (2.4%; 1/41) patient had a ECOG PS score of 1. Regarding the FIGO stage at initial diagnosis, 4.9% (2/41) of patients were stage I, 61.0% (25/41) were stage II, 19.5% (8/41) were stage III, and 14.6% (6/41) were stage IV. All patients had received at least two prior lines of therapy. Twenty-six patients (63.4%; 26/41) had received more than two previous therapies. The median time since the last treatment was 6.1 months (range 0.7–44.4 months).

**Table 1:** Baseline patient characteristics.

Characteristic	N (%)
Age (years)	
Median	53 ± 10.9
Histology	
Squamous cell carcinoma	34 (82.9)
Adenocarcinoma	6 (14.6)
Other (Small cell neuroendocrine carcinoma)	1 (2.4)
ECOG PS	
0	40 (97.6)
1	1 (2.4)
FIGO stage at initial diagnosis	
I	2 (4.9)
II	25 (61.0)
III	8 (19.5)
IV	6 (14.6)
Prior treatment	
Chemotherapy	33 (80.5)
Radiotherapy	29 (70.7)
Radiotherapy and Chemotherapy	28 (68.3)
Prior lines for therapies	
<2	15 (36.6)
>2	26 (63.4)
Interval between last treatment and progression (Months)	
Median	6.1
Range	0.7-44.4
Sites of recurrence	
Within the irradiated field	27 (65.9)
Outside the irradiated field	10 (24.4)
Within and outside the irradiated field	4 (9.7)

Data are presented as No. (%) unless otherwise indicated.

ECOG PS, Eastern Cooperative Oncology Group performance status; FIGO, International Federation of Gynecology and Obstetrics.

### Efficacy

**Figure 2:**
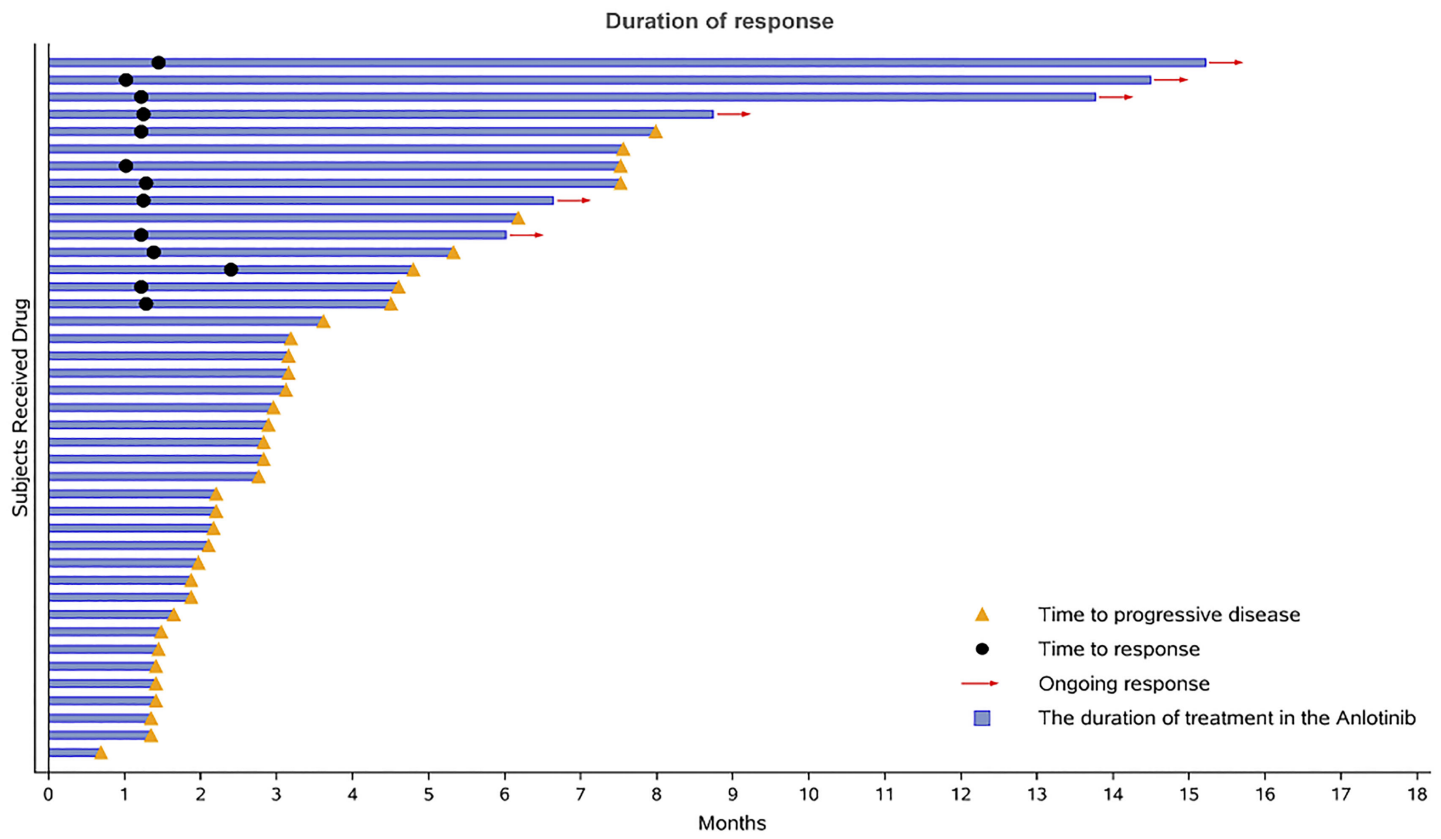
Duration of responses. The length of each bar represents the duration of treatment for each patient.

**Figure 3:**
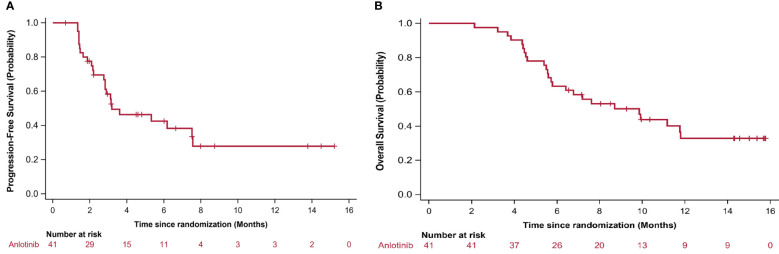
Kaplan–Meier curves for **(A)** progression-free survival (PFS) and **(B)** overall survival (OS). PFS and OS was assessed in all patients.

During the 16-month follow-up period, 10 (24.4%) patients had a confirmed response to treatment, of which 6 (14.6%) patients had an ongoing response. The time to response occurred within 2.3 months (**Figure 2**). The median PFS was 3.2 (95% CI: 0.4–5.9) months in patients with anlotinib treatment (**Figure 3A**), and the median OS was 9.9 (95% CI: 5.7-11.8) months in patients with anlotinib treatment (**Figure 3B**). Forty (40/41) patients were evaluated for treatment response. No patient achieved CR and 10 (24.4%) patients achieved PR. The ORR was 24.4% (95% CI: 12.4–40.3). In addition, 14 (34.2%) patients were confirmed to have SD and 16 (39.0%) patients had confirmed PD. The DCR was 58.5% (95% CI: 42.1–73.7; **Table 2**). Subgroup analysis for the ORR in stratified clinicopathological features revealed that tumor regressions were observed in all subgroups, and overall ORR was consistent among subgroups (**Table 3**).

**Table 2:** Overall tumor response in patients.

Parameter	N (%)
Complete Response (CR)	0 (0)
Partial Response (PR)	10 (24.4)
Stable Disease (SD)	14 (34.1)
Progressive Disease (PD)	16 (39.0)
no-evaluation (NE)	1 (2.4)
ORR (CR+PR), 95%CI	10 (24.4), 12.4-40.3
DCR (CR+PR+SD), 95%CI	24 (58.5), 42.1-73.7

Data are presented as No. (%) unless otherwise specified. Responses were assessed in accordance with RECIST version 1.1. Only confirmed responses were included.

ORR, overall response rate; DCR, disease control rate; CI, confidence interval.

**Table 3:** Subgroup analysis for ORR in patients treated with Anlotinib.

Features	ORR	95%CI	RR	95%CI	P
**Age (years)**					
> 53	11.76	1.46-36.44	3.75	0.68-20.57	0.128
≤ 53	33.33	15.63-55.32			
**Histological type**					
Squamous cell carcinoma	26.47	12.88-44.36	0.46	0.05-4.39	0.502
Non-Squamous cell carcinoma	14.29	0.36-57.87			
**FIGO stage**					
I and II	22.22	8.62-42.26	1.40	0.32-6.10	0.654
III and IV	28.57	8.39-58.10			
**Prior treatment**					
Radiotherapy and chemotherapy	25.93	11.11-46.28	0.78	0.17-3.63	0.751
Radiotherapy or chemotherapy	21.43	4.66-50.80			
**Sites of recurrence**					
Within the irradiated field	32.14	15.88-52.35	0.18	0.02-1.57	0.120
Outside the irradiated field	7.69	0.19-36.03			
**Type of recurrence**					
Organ	21.43	8.30-40.95	1.63	0.37-7.19	0.520
Lymph nodes	30.77	9.09-61.43			

CI, confidence interval; FIGO, International Federation of Gynecology and Obstetrics; RR, risk ratio; ORR, objective response rate.

### Safety

By the cutoff date of April 2020, all patients were evaluable for toxicity. In general, anlotinib was well tolerated. The adverse events (AEs) are listed in **Table 4**. All patients experienced AEs. The majority of AEs were grade 1 or 2. The most common adverse events were anemia (29.3%), hand-foot syndrome (26.8%), lymphocytopenia (24.4%), urinary leukocyte positivity (22%), fatigue (19.5%), and hematuria (17.1%). The high-grade AEs (grade 3) included urinary leukocyte positivity (9.8%), hematuria (4.9%), and hypertension (2.4%). No patient had grade 4 AEs. Neither unexpected safety signals nor treatment-related death occurred.

**Table 4:** Anlotinib-related adverse events.

Adverse Events	All GradeN (%)	Grade 1N (%)	Grade 2N (%)	Grade 3N (%)
Anemia	12 (29.3)	9 (22.0)	3(7.3)	0 (0)
Hand-foot Syndrome	11 (26.8)	10 (24.4)	1 (2.4)	0 (0)
Lymphocytopenia	10 (24.4)	7 (17.1)	3 (7.3)	0 (0)
Urinary Leukocyte Positive	9 (22.0)	4 (9.8)	1 (2.4)	4 (9.8)
Fatigue	8 (19.5)	8 (19.5)	0 (0)	0 (0)
Hematuria	7 (17.1)	2 (4.9)	3 (7.3)	2 (4.9)
Proteinuria	7 (17.1)	0 (0)	7 (17.1)	0 (0)
Hyperuricemia	5 (12.2)	5 (12.2)	0 (0)	0 (0)
Lactic Dehydrogenase Elevation	5 (12.2)	5 (12.2)	0 (0)	0 (0)
Diarrhea	5 (12.1)	5 (12.1)	0 (0)	0 (0)
Cough	4 (9.8)	4 (9.8)	0 (0)	0 (0)
Hypertension	4 (9.8)	3 (7.3)	0 (0)	1 (2.4)
Leucopenia	4 (9.8)	0 (0)	4 (9.8)	0 (0)
Increased Creatinine	4 (9.8)	4 (9.8)	0 (0)	0 (0)
Urea Elevation	4 (9.8)	4 (9.8)	0 (0)	0 (0)

## Discussion

Patients with recurrent or metastatic CC always exhibit weak responses to palliative chemotherapy and poor prognoses. Novel targeted treatment that prolongs survival without inducing toxicity is paramount. This is the first study to evaluate the efficacy and safety of anlotinib monotherapy in patients with recurrent or metastatic CC who had previously received more than two lines of treatment. Forty-one patients with advanced CC were included. The ORR was 24.4% (95% CI = 12.4–40.3), and the toxicity was controllable. To our knowledge, this study is the first phase II trial to reveal a survival benefit in patients with recurrent or metastatic advanced cervical cancer.

For recurrent CC, palliative chemotherapy remains the main treatment, but it does not provide satisfactory survival benefits (25). A phase III study of cisplatin monotherapy in stage IVB of CC revealed that the median PFS was 2.8 months (26). A phase II evaluation of oxaliplatin in previously treated cervix carcinoma demonstrated a response rate of only 8.3% (27). A phase II evaluation of topotecan as a single-agent second-line therapy in persistent or recurrent carcinoma of the cervix illustrated that the median PFS was only 2.4 months (28). Patients with CC have limited treatment options after recurrence or metastasis, especially advanced patients who are unresponsive to second-line or later treatment (29). Thus, once recurrence or metastasis occurs, it is difficult to obtain clinical benefits through chemotherapy alone. Therefore, it is necessary to provide new treatment methods for patients with advanced CC.

Sufficient clinical studies have confirmed that anti-angiogenic therapy is an effective treatment for solid tumors. Bevacizumab, a related drug that inhibits tumor angiogenesis, has been clinically approved for use. The Gynecologic Oncology Group (GOG) 227C trial revealed that bevacizumab monotherapy produced significant improvements in activity, and it was well-tolerated in patients with recurrent CC (30). The GOG 240 trial revealed the survival benefit of bevacizumab in combination with chemotherapy in patients with refractory, recurrent, or metastatic CC (15). The median OS was 17 months, and the median PFS was 8.2 months. However, combined treatment always led to major AEs and poor maintenance. In another phase II trial of sunitinib monotherapy in advanced or metastatic CC, no patients achieved CR and the median DFS was 3.5 months (31). A phase II study compared the efficacy of pazopanib and lapatinib monotherapies as a second-line or above therapy for advanced or recurrent CC (32). The results revealed that the median PFS in the pazopanib group (n=74) and the lapatinib group (n=78) were 18.1 weeks and 17.1 weeks, respectively (P<0.05). However, there was no statistically significant difference in OS between the groups. Pembrolizumab, an immune checkpoint inhibitor, is the recommended second-line treatment for recurrent or metastatic advanced CC. However, in the KEYNOTE-158 phase II clinical trial, the ORR of pembrolizumab in patients with advanced CC was only 13% (16% in PD-L1–positive patients) (33), which was much lower than that of our study (24.4%). Compared with the findings for other targeted drugs in the GOG Phase II trials, the 3.2-month median PFS of anlotinib is similar to that of other targeted monotherapies (34–37). The reason for the lower PFS than that of bevacizumab might be related to the synergistic benefits of bevacizumab in combination with chemotherapy. Although the median PFS in this study was relatively low, the disease control rate was high and the time to response was short, with the benefit of delaying disease progression.

Anlotinib, as a new type of oral multi-target TKI, has been confirmed to inhibit angiogenesis and reduce microvessel density in *in vivo* experiments (38). Compared with other receptor tyrosine kinase inhibitors, anlotinib inhibits more targets, including VEGFR1-3, c-Kit, PDGFR-α, and FGFR1–3. More importantly, according to the results of previous controlled studies, the anti-angiogenic effect of anlotinib is stronger than those of three commonly used anti-angiogenic drugs (sorafenib, sunitinib, and pazopanib) (39). Preclinical data illustrated that anlotinib treatment alone achieved the same effect as the combination of anlotinib and chemotherapy in the suppression of tumor growth and neoangiogenesis (40).

There are sparse clinical data on anlotinib in the treatment of CC. In the present study, we achieved an ORR of 24.4%, the DCR was 58.5%, and median PFS was 3.2 months. This shows that anlotinib has a certain anti-tumor effect in recurrent or metastatic advanced CC, and it can prolong the survival of patients compared with other monotherapies in recurrent or metastatic advanced CC (9). With the extension of follow-up, we believe that the ongoing response time will be further extended. During the 16-month follow-up of our study, no treatment-related death occurred. Compared with other chemotherapeutic drugs, anlotinib exhibits a more prominent advantage in its oral dosage; therefore, it may be a convenient and economical medication for recurrent or metastatic advanced CC.

Anlotinib was well tolerated in patients, and the most common AEs were anemia (29.3%), hand–foot syndrome (26.8%), lymphocytopenia (24.4%), urinary leukocyte positivity (22%), and fatigue (19.5%). Most AEs were grade 1 or 2. High-grade AEs (grade 3) included urinary leukocyte positivity (9.8%), hematuria (4.9%), and hypertension (2.4%). A previous study demonstrated that the rate of grade 3 AEs of lapatinib and pazopanib (both are multi-targeted tyrosine kinase inhibitors) in patients with recurrent or metastatic CC were 32% and 42%, respectively, and the rates of grade 4 AEs were 9% and 12%, respectively (32). In the GOG 227C study, the most frequent grade 3 AE of bevacizumab monotherapy was hypertension (15.2%), and 10.9% of patients experienced grade 4 AEs. In the chemotherapy–bevacizumab group in the GOG-240 study, the rate of grade 3 or 4 hematologic toxicities was 58% for neutropenia and 36% for leukopenia (15). In the carboplatin–paclitaxel group in the JCOG-0505 study, the rates of grade 3 or 4 hematologic toxicities were 76.2% for neutropenia, 44.4% for anemia, and 24.6% for thrombocytopenia (41). Compared with these monotherapy and combination regimens, the AEs of anlotinib monotherapy were significantly reduced. Neither unexpected safety signals nor treatment-related death occurred. The only death was attributable to the continuous progression of tumor lesions, causing perforation of the digestive tract, which was not related to anlotinib. Anlotinib therefore provides potential options for patients with advanced disease for whom palliative chemotherapies have failed.

In conclusion, as the first phase II trial of anlotinib in recurrent or metastatic advanced CC, this study had some limitations. This was a single-arm phase II trial, and head-to-head comparisons of anlotinib and chemotherapy are needed to identify the clinical outcomes. A limited number of subjects were included in this trial. However, anlotinib monotherapy may have favorable efficacy and safety in relapsed and metastatic CC. In the future, a phase III randomized controlled trial is necessary to validate the findings of this study.

## Data Availability Statement

The original contributions presented in the study are included in the article/supplementary material. Further inquiries can be directed to the corresponding authors.

## Ethics Statement

The studies involving human participants were reviewed and approved by Institutional Ethics Committees of Fudan University Shanghai Cancer Center. The patients/participants provided their written informed consent to participate in this study.

## Author Contributions

Data collection: JZ, GK, and CS. Case selection: JZ, GK, ZZ, XW. Evaluation: JZ, GK, YC, and LX. Statistical analysis: JZ, GK, and CS. Paper writing: JZ, GK, and XW. All authors contributed to the article and approved the submitted version.

## Conflict of Interest

The authors declare that the research was conducted in the absence of any commercial or financial relationships that could be construed as a potential conflict of interest.

## Publisher’s Note

All claims expressed in this article are solely those of the authors and do not necessarily represent those of their affiliated organizations, or those of the publisher, the editors and the reviewers. Any product that may be evaluated in this article, or claim that may be made by its manufacturer, is not guaranteed or endorsed by the publisher.
